# GO-INO: Graph Optimization MEMS-IMU/NHC/Odometer Integration for Ground Vehicle Positioning

**DOI:** 10.3390/mi13091400

**Published:** 2022-08-26

**Authors:** Kai Zhu, Yating Yu, Bin Wu, Changhui Jiang

**Affiliations:** 1 School of Automobile and Traffic Engineering, Jiangsu University of Technology, Changzhou 213001, China; 2 School of Mechanical, Electronic and Control Engineering, Beijing Jiaotong University, Beijing 100044, China; 3 Department of Remote Sensing and Photogrammetry, Finnish Geospatial Research Institute, Masala, FI-0245 Espoo, Finland

**Keywords:** GNSS, INS, signal outage, factor graph optimization

## Abstract

Global navigation satellite system (GNSS) and inertial navigation system (INS) are indispensable for ground vehicle position and navigation. The Kalman filter (KF) is the first choice to integrate them and output more reliable navigation solutions. However, the GNSS signal is denied in urban areas, i.e., tunnels, and the INS position errors diverge quickly over time. Under normal conditions, the ground vehicle will not slide or jump off the ground; nonholonomic constraints (NHC) and odometers are available to aid the INS and reduce its position errors. Factor graph optimization (FGO) recently attracted attention as an advanced sensor fusion algorithm. This paper implemented the FGO method based on GNSS/INS/NHC/Odometer integration. In the FGO, state transformation, measurement model, the NHC, and the odometer were all regarded as constraints employed to construct a graph; an iterative process was utilized to find the optimal estimation results. Two experiments were carried out: firstly, the FGO-GNSS/INS performance was assessed and compared with the KF-GNSS/INS; secondly, we compared the FGO-GNSS/INS/NHC/Odometer and KF-GNSS/INS/NHC/Odometer under GNSS denied environments. Experimental results supported that the FGO improved the performance.

## 1. Introduction

With the booming of the location-based service (LBS), i.e., autonomous driving, unmanned aerial vehicles (UAV), reliable positioning results are critical for these applications [1,2,3,4,5]. GNSS (global navigation satellite system) is the dominant tool for providing precise and dependable three-dimensional positioning information. In the open-sky environments [6], there are usually enough in-view satellites, and the GNSS receivers can generate exact three-dimensional positions. However, GNSS signals might be obstructed in urban areas with tall buildings or reflected by the surrounded buildings. The signal blockage will degrade the geometry distribution of the in-view satellites, and the positioning accuracy will decrease [7,8]. Therefore, GNSS is usually integrated with the INS to generate more reliable navigation solutions [9,10].

INS is a self-contained navigation system that generates position, velocity, and attitude (PVA) information through processing the gyroscope and raw accelerometer measurements from the inertial measurement unit (IMU) [11,12,13]. Due to the complex noises contained in the basic measurements from the IMU, INS positioning errors diverge quickly over time. In the GNSS/INS-integrated navigation system, the integration filter, usually the Kalman filter (KF), estimates the IMU error states and compensates for its errors; in this manner, the INS can still output precise navigation solutions in a short time during GNSS signal outage [14,15]. In urban areas, the GNSS signal is usually blocked by surrounding buildings. Improving the INS accuracy during GNSS signal outages is significant [16,17,18].

The methods to improve the INS positioning accuracy could be categorized into two groups. The first method is to analyze and denoise the raw measurements of the IMU. Allan variance was first employed in the IMU measurements noise analysis, modeling, and quantification [13,14,15]. Then, an autoregressive moving average model (ARMA) method was employed in estimating and modeling the IMU noises [19,20]. Additionally, some variants of the ARMA were proposed, e.g., a Sage Husa adaptive robust Kalman filtering method was utilized to tune the parameters of the ARMA [20]; an adaptive unscented fading Kalman filter for reducing drift of fiber optic gyroscope [21]; and a robust Kalman filter was proposed for gyro random noise [22]. In addition, wavelet denoising methods and machine learning methods were also employed in the IMU raw measurements denoising. Naser first proposed a wavelet denoising for IMU alignment [11]; after this, some other research was carried out to further explore the IMU denoising with different wavelet denoising methods [23,24,25]. Machine learning methods, e.g., support vector machine (SVM) [26], long-short-term-memory recurrent neural networks (LSTM-RNN) [27], deep simple reduce unit recurrent neural network (SRU-RNN) [28], and a neural architecture search neural network were employed in the MEMS IMU denoising [29].

In addition to the IMU raw measurements noising, modifications of the GNSS/INS integration filter were carried out to decrease the INS navigation solution errors. Some machine learning methods, radial basis function neural network, LSTM, recurrent fuzzy wavelet neural networks, and deep reinforcement learning were trained while the GNSS/INS integration worked well; they were employed to compensate for the INS navigation solution errors during signal outages [30,31,32,33,34]. For ground vehicles, some unique characteristics were extracted and utilized to suppress the INS navigation solution errors [35,36,37,38,39]. While the ground vehicle drove steadily on the road, the up-direction and lateral velocity were almost zero. The nonholonomic constraints (NHC) decreased the INS positioning errors [35,36,37,38,39]. In addition, an odometer was usually installed in the ground vehicles. Therefore, GNSS/INS/Odometry/NHC was constructed and assessed under GNSS signal-challenging environments [36]. 

Factor graph optimization (FGO) recently attracted attention as an alternative method to GNSS/INS integration [40,41,42,43]. Wen et al., presented comprehensive comparisons between the KF-GNSS/INS and the FGO-GNSS/INS with ground vehicle experiments in urban areas [40]. The FGO relinearization and the full use of the historical time-correlation measurements contribute to the FGO-GNSS/INS’s better performance than the KF-GNSS/INS [40]. Li et al., implemented the FGO-GNSS/INS tight integration, presenting the FGO-GNSS/INS improvements to the KF-GNSS/INS [41]. Researchers from the Georgia Institute of Technology released the optimization library GTSAM and implemented the FGO-GNSS/INS; FGO’s better performance than the KF-GNSS/INS is demonstrated [42]. This paper implemented the FGO-GNSS/INS integration and the FGO-INS/NHC/Odometer integration in GNSS-denied environments. FGO handles the measurements as constraints, making full use of the historical information and the inherent relationship among the states. Moreover, it is convenient to delete or add measures or constraints. Two field tests were carried out in urban areas to assess the performance of the FGO-GNSS/INS/Odometer integration and the FGO-INS/NHC/Odometer integration. 

The remainder of the paper is organized as follows: Section 2 gives the KF-GNSS/INS/ Odometer details and the NHC mechanisms, state propagation model, measurement model, and the KF are presented; Section 3 presents the FGO-GNSS/INS integration and the FGO-INS/NHC/Odometer integration; Section 4 presents the field tests, the experimental results, and analysis. Finally, conclusions are drawn, and some discussions are also listed to deepen this method.

## 2. KF-GNSS/INS/NHC Integration

**Figure 1:**
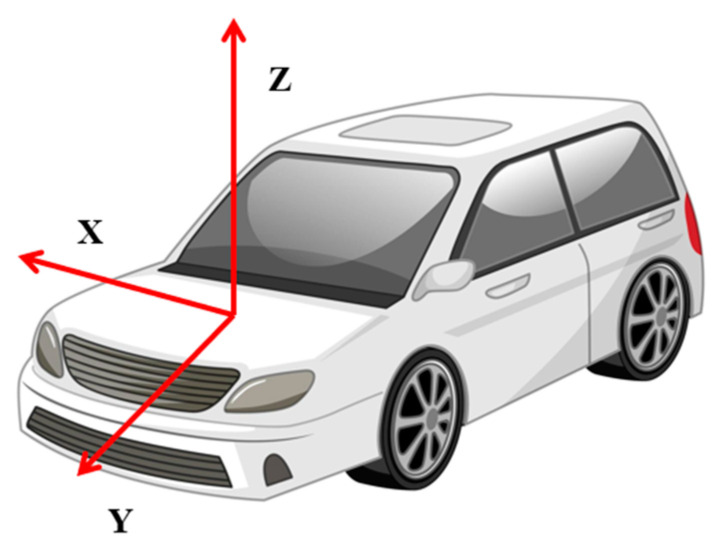
Ground vehicle body coordinates.

While the ground vehicles usually drive on the road, we can find two motion characteristics in the vehicle body coordinates (1) up-direction velocity is approximately zero; (2) velocity of the lateral is approximately zero. The equations are written as [44]:

(1) $$\left\{\begin{array}{c} v_{x}^{v}\approx 0\\ v_{z}^{v}\approx 0 \end{array}\right.$$

where $v_{x}^{v}$ denotes the lateral velocity in the vehicle body coordinates, $v_{z}^{v}$ indicates the up-direction velocity. The ground vehicle coordinate is presented in Figure 1. X axis of the vehicle body coordinate is defined as pointing to the right side of the vehicle body, the Y axis of the vehicle body coordinate is defined as pointing to the direction of the vehicle heading, the Z axis of the vehicle body coordinate is defined as pointing to the direction of the roof.

While the ground vehicle drives smoothly on the road, these velocity constraints can be employed to suppress the diverging positioning errors of the INS. The motion constraints can be used as measurements in the GNSS/INS integration filter. While the IMU is installed in the vehicle for sensing the motions, there is a misalignment angle between the IMU body coordinate and the vehicle body coordinate. While applying the movement constraints in the integration filter, the measurement vector should be projected to the IMU body coordinate through the conversion matrix calculated through the misalignment angle. Here, assuming the misalignment matrix is $C_{v}^{b}$, then the conversion can be modeled as:

(2) $$V^{b}=C_{v}^{b}\cdot V^{v}$$

(3) $$V^{b}=\left[\begin{array}{c} v_{x}^{b}\\ v_{y}^{b}\\ v_{z}^{b} \end{array}\right]=C_{v}^{b}\left[\begin{array}{c} v_{x}^{v}\\ v_{y}^{v}\\ v_{z}^{v} \end{array}\right]=\left[\begin{array}{c} \sin\alpha_{\psi }\cos\alpha_{\theta }\\ \cos\alpha_{\psi }\cos\alpha_{\theta }\\ \sin\alpha_{\theta } \end{array}\right]\cdot v_{y}^{v}$$

where $\alpha_{\psi }$ denotes the misalignment heading angle, $\alpha_{\theta }$ denotes the pitch misalignment angle, $V^{b}$ denotes the velocity vector in the IMU body coordinate, $v_{x}^{b}$, $v_{y}^{b}$ and $v_{z}^{b}$ denote the three-axis velocity in the IMU body coordinate, $V^{v}$ denotes the velocity vector in the vehicle body frame, and $v_{x}^{v}$, $v_{y}^{v}$ and $v_{z}^{v}$ denote the three-axis velocity in the vehicle body coordinate.

### 2.1. State Propagation and Measurement Model

In the KF-GNSS/INS integration, attitude errors, velocity errors, position errors, gyroscope three-axis bias, and accelerometer three-axis bias are included in the state vector. The integration filter estimates these state variables, which is employed for compensating the INS errors. Assuming the state vector is termed as $X_{INS}$.

(4) $$X_{INS}={\left[\delta \phi ,\delta vel,\delta pos,\delta \varepsilon ,\delta \nabla \right]}^{T}$$

where $\delta \phi =\left[\alpha ,\beta ,\gamma \right]$ refers to the three-axis attitude errors (pitch, roll, and yaw angle errors), $\delta vel=\left[\delta v_{E}\;\delta v_{N}\;\delta v_{U}\right]$ means the velocity vector, which is composed of east, north, and up velocity error (East-North-Up coordinates), $\delta pos=\left[\delta L\;\delta \lambda \;\delta H\right]$ is the position error vector which is composed of latitude, longitude, and height error, then, $\delta \varepsilon =\left[\varepsilon_{x}\;\varepsilon_{y}\;\varepsilon_{z}\right]$ denotes the three-axis accelerometer bias errors, and $\delta \nabla =\left[\nabla_{x}\;\nabla_{y}\;\nabla_{z}\right]$ refers to the three-axis gyroscope bias errors, respectively. 

The state transformation model of the GNSS/INS integration model is built based on the INS error propagation; the state equation is written as

(5) $$X_{k+1}^{}=F_{k,k+1}\cdot X_{k}^{}+w$$

where $X_{k}^{I}$ denotes the state vector, $F_{k,k+1}$ represents the state transformation matrix, and w indicates the state model noise matrix [35].

In the GNSS/INS integration, the position and velocity from GNSS and INS are subtracted and then employed to compose the measurement vector of the integration filter. The measurement vector is written as:

(6) $$Z_{k+1}=H_{k+1}X_{k+1}+\mu$$

(7) $$Z_{k+1}=\left[\begin{array}{l} \left(L_{k+1}^{INS}-L_{k+1}^{GNSS}\right)\cdot \left(R_{M}+h\right)\\ \left(\lambda_{k+1}^{INS}-\lambda_{k+1}^{GNSS}\right)\cdot \left(R_{N}+h\right)\cdot \cos\left(L\right)\\ h_{k+1}^{INS}-h_{k+1}^{GNSS}\\ v_{E,k+1}^{INS}-v_{E,k+1}^{GNSS}\\ v_{N,k+1}^{INS}-v_{N,k+1}^{GNSS}\\ v_{U,k+1}^{INS}-v_{U,k+1}^{GNSS} \end{array}\right]$$

where the measurement noise, the superscript “INS” and “GNSS” denote the information from the INS and GNSS, respectively, $Z_{k+1}$ indicates the measurement vector, which is composed of the position and velocity difference between the GNSS and INS (Equation (7)), μ denotes measurement noise, $H_{k+1}$ indicates the observation matrix and it is written as

(8) $$Z_{k+1}=\left[\begin{array}{ccc} diag\left[R_{M}+h\cdot \left(R_{N}+h\right)\cos\left(L\right)\;1\right] & 0_{3\times 3} & 0_{3\times 3}\\ 0_{3\times 3} & & 0_{3\times 3} \end{array}\right]X_{k+1}+\mu$$

### 2.2. NHC Measurement Model

As aforementioned, while the vehicle drives smoothly on the road, the up and lateral velocity in the vehicle body coordinates are both almost zero and are termed as $v_{x}^{b}$ and $v_{z}^{b}$. Converting the speed from the vehicle body frame to the IMU body frame:

(9) $${\left(V_{NHC}\right)}_{b}^{I}=C_{b}^{I}\cdot {\left(V_{NHC}\right)}^{b}$$

where $C_{b}^{I}$ denotes the velocity conversion matrix, ${\left(V_{NHC}\right)}_{b}^{I}$ denotes the converted velocity in the IMU body coordinates; and then, converting the velocity from the IMU frame to the navigation frame and the procedure is expressed as

(10) $${\left(V_{NHC}\right)}_{I}^{n}=C_{I}^{n}\cdot {\left(V_{NHC}\right)}_{b}^{I}$$

where $C_{I}^{n}$ denotes the velocity conversion matrix, and the ${\left(V_{NHC}\right)}_{b}^{I}$ and ${\left(V_{NHC}\right)}_{I}^{n}$ denote the velocity in the IMU body frame and the navigation frames. The NHC measurement vector is written as

(11) $$Z_{k+1}^{NHC}={\left(V_{NHC}\right)}_{I}^{n}-V_{n}^{INS}$$

$V_{n}^{INS}$ denotes the velocity obtained from processing the IMU raw measurements.

### 2.3. Odometer Measurement Model

An odometer can provide odometry measurements of the ground vehicle, and the ground vehicle velocity can be estimated with the odometry difference. The odometer output is written as

(12) $${\left(V_{Odo}\right)}^{b}={\left[\begin{array}{ccc} 0 & V_{odo,y}^{b} & 0 \end{array}\right]}^{T}$$

The odometer velocity is measured in IMU body coordinates; we assumed the misalignment angle between the odometer and the IMU is zero. Then, converting the velocity from the IMU body frame to the East-North-Up navigation coordinates, the measurement vector is written as

(13) $$Z_{k+1}^{Odo}=C_{b}^{n}\cdot {\left(V_{Odo}\right)}^{b}-V_{n}^{INS}=\left[\begin{array}{c} V_{odoE}^{n}-V_{IE}\\ V_{odoN}^{n}-V_{IN}\\ V_{odoU}^{n}-V_{IU} \end{array}\right]=H_{Odo}\cdot X+V_{Odo}$$

where $V_{n}^{INS}$ denotes the IMU velocity, ${\left(V_{Odo}\right)}^{b}$ denotes the velocity from the odometer, $H_{Odo}$ denotes the observation matrix, and $V_{Odo}$ denotes the measurement noise vector.

(14) $$H_{Odo}=\left[\begin{array}{ccccc} 0_{1\times 3} & 1 & 0 & 0 & 0_{1\times 9}\\ 0_{1\times 3} & 0 & 1 & 0 & 0_{1\times 9}\\ 0_{1\times 3} & 0 & 0 & 1 & 0_{1\times 9} \end{array}\right]$$

### 2.4. Kalman Filter

State and measurement models of the KF-GNSS/INS integration methods are listed in Equations (4)–(11); the KF is usually employed to estimate the state vector recursively. In the typical KF, five equations are generally essential for the two steps in KF: predicting and updating procedures. 

In the prediction step, KF predicts the state vector through the state propagation model, and the state estimation errors covariance matrix is also predicted through the state propagation matrix. The equations of the prediction are written as

(15) $$X_{k}^{-}=F_{k|k-1}X_{k-1}$$

(16) $$P_{k}^{-}=F_{k|k-1}P_{k-1}F_{k|k-1}^{T}+Q_{k-1}$$

The state vector, state estimation errors covariance matrix, and the Kalman gain matrix are updated in the updating step. The equations are written as

(17) $$K_{k}=P_{k}^{-}\cdot H_{k}^{T}{\left(H_{k}\cdot P_{k}^{-}\cdot H_{k}^{T}+R_{k}\right)}^{-1}$$

(18) $${\hat{X}}_{k}=X_{k}^{-}+K_{k}\cdot \left(Z_{k}-H_{k}\cdot X_{k}^{-}\right)$$

(19) $$P_{k}=\left(I-K_{k}\cdot H_{k}\right)\cdot P_{k}^{-}$$

where $F_{k|k-1}$ denotes the state transformation matrix, Q denotes the covariance matrix of the state process noise, and R denotes the covariance matrix of the measurement noise, P denotes the state estimation errors covariance matrix, H denotes the observation matrix, “−”denotes the prediction, “^”denotes the estimation, and K denotes the Kalman gain matrix. While the GNSS is expected, the system works with GNSS/INS integration mode, and reliable navigation solutions are expected. At the same time, GNSS is denied; according to the vehicle state detection results, NHC and odometer measurements are added to construct the integration to suppress the INS position errors.

## 3. FGO-GNSS/INS/NHC/Odometer

Recently, factor graph optimization (FGO) has attracted much attention in multi-sensor fusion [42,43]; the FGO utilizes the graphic model to represent the states and the measurements [43]. Unlike the KF, the FGO encodes the historical state transformation and measurements as the factors. It estimates the set of states at each epoch through a Gauss–Newton method or Levenberg–Marquardt (LM) method. In the FGO, the edge connects the factor node and the variable node. The factorized function is written as [13,14]:

(20) $$f\left(\chi \right)=\prod_{i}f_{i}\left(\chi_{i}\right)$$

where $\chi_{i}$ denotes nodes of the state variables. In the FGO, the optimal estimates $\hat{\chi }$ are obtained by minimizing the errors of the entire graph, and the procedure is summarized as

(21) $$\hat{\chi }=\arg\underset{\chi }{\min}\left(\prod_{i}f_{i}\left(\chi_{i}\right)\right)$$

**Figure 2:**
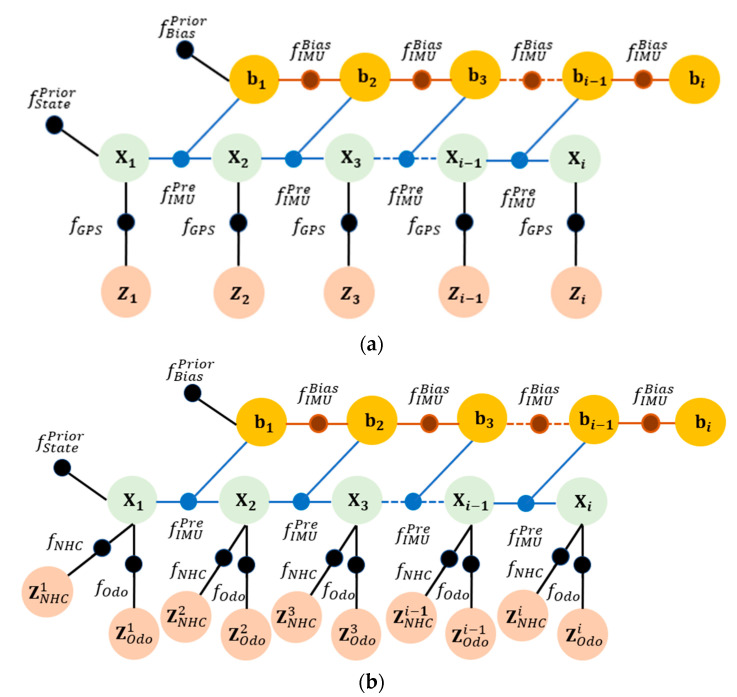
FGO GNSS/INS/NHC/Odometer integration. (**a**) FGO-GNSS/INS. (**b**) FGO-IMU/ Odometer/NHC.

Here, there are four types of measurements: GNSS measurements, INS measurements, NHC measurements, and the odometer measurements. Suppose the process noise and the measurement noise are both subject to zero-mean Gaussian distributions with covariance matrix $\Sigma_{k}$ and $\Lambda_{k}$. The optimal estimation of the state variables can be expressed as

(22) $$\hat{X}=\arg\max\prod P\left(Z_{i}\left|X_{i}\right.\right)\prod P\left(X_{i}\left|X_{i-1},u_{i}\right.\right)$$

(23) $$P\left(Z_{i}\left|X_{i}\right.\right)\propto \exp\left(-\frac{1}{2}{\left\|h_{i}\left(X_{i-1,}u_{i}\right)-X_{i}\right\|}_{\Sigma_{i}}^{2}\right)$$

(24) $$P\left(Z_{i}\left|X_{i}\right.\right)\propto \exp\left(-\frac{1}{2}{\left\|h_{i}\left(X_{i-1}\right)-X_{i}\right\|}_{\Lambda_{i}}^{2}\right)$$

where $Z_{i}$ refers to the measurement vector, $u_{i}$ refers to the control input, and $\hat{X}$ refers to the estimates of the state vector. Then, the optimal state estimates of the state can be converted to a nonlinear square problem, and the optimal state can be obtained by minimizing the cost function, which is written as:

(25) $$\hat{X}=\arg\min\left(\sum_{i=1}^{K}{\left\|f_{i}\left(X_{i-1,}u_{i}\right)-X_{i}\right\|}_{\Sigma_{i}}^{2}+\sum_{i=1}^{K}{\left\|h_{i}\left(X_{i-1}\right)-X_{i}\right\|}_{\Lambda_{i}}^{2}\right)$$

where $\left\|\cdot \right\|$ means the Mahalanobis norm. Here, there are two different conditions, and Figure 2 presents the factor graph structure for the GNSS/INS integration (Figure 2a) and INS/NHC/Odometer integration (Figure 2b). The following subsections present the IMU, NHC, and odometer factors. 

### 3.1. IMU Preintegration Factor

A standard IMU contains a three-axis accelerometer and gyroscope, which measures the acceleration and the rotation. The IMU measurement model is written as:

(26) $${\tilde{\omega }}_{I}\left(t\right)=\omega_{WI}\left(t\right)+b_{g}\left(t\right)+\eta_{g}\left(t\right)$$

(27) $${\tilde{a}}_{I}\left(t\right)=R_{WI}^{T}\left(t\right)\left(a_{W}\left(t\right)-g_{W}\right)+b_{a}\left(t\right)+\eta_{a}\left(t\right)$$

where ${\tilde{\omega }}_{I}\left(t\right)$ denotes the angular velocity in the IMU coordinates, $\omega_{WI}\left(t\right)$ denotes the angular velocity of the IMU frame relative to the world frame, $b_{g}\left(t\right)$ denotes the angular rate time-varying bias, $\eta_{g}\left(t\right)$ denotes the Gaussian white noise, ${\tilde{a}}_{I}\left(t\right)$ denotes the accelerometer measurements in the IMU frame, $R_{WI}^{T}\left(t\right)$ denotes the rotation matrix from the world frame to the IMU frame, $a_{W}\left(t\right)$ and $g_{W}$ denote the acceleration measurements and the gravity vector in the world frame, and $b_{a}\left(t\right)$ and $\eta_{a}\left(t\right)$ denote the angular rate time-varying bias and the Gaussian white noise. 

The IMU kinetic model is written as:

(28) $${\dot{R}}_{WI}=R_{WI}\omega_{WI}^{\wedge }$$

(29) $${\dot{V}}_{W}=a_{W}$$

(30) $${\dot{P}}_{W}=v_{W}$$

where $\omega^{\wedge }$ is written as

(31) $$\omega^{\wedge }={\left[\begin{array}{l} \omega_{1}\\ \omega_{2}\\ \omega_{3} \end{array}\right]}^{\wedge }=\left[\begin{array}{lll} 0 & \omega_{3} & \omega_{2}\\ \omega_{3} & 0 & \omega_{1}\\ -\omega_{2} & -\omega_{1} & 0 \end{array}\right]$$

Suppose that the angular rate and acceleration remain invariant during the IMU measurement updating period $\left(t,t+\Delta t\right)$. The discrete form of the motion model is written as:

(32) $$R_{WI}\left(t+\Delta t\right)=R_{WI}\left(t\right)\mathrm{Exp}\left[\left({\tilde{\omega }}_{I}\left(t\right)-b_{g}\left(t\right)-\eta_{gd}\left(t\right)\right)\Delta t\right]$$

(33) $$v_{W}\left(t+\Delta t\right)=v_{W}\left(t\right)+g_{W}\Delta t+R_{WI}\left(t\right)\left[{\tilde{a}}_{I}\left(t\right)-b_{a}\left(t\right)-\eta_{ad}\left(t\right)\right]\Delta t$$

(34) $$P_{W}\left(t+\Delta t\right)=P_{W}\left(t\right)+v_{W}\left(t\right)P_{W}\left(t+\Delta t\right)+\frac{1}{2}g_{W}\Delta t^{2}+\frac{1}{2}R_{WI}\left[{\tilde{a}}_{I}\left(t\right)-b_{a}\left(t\right)-\eta_{ad}\left(t\right)\right]\Delta t^{2}$$

The IMU factor can be written as

(35) $$x_{k}=h^{INS}\left(x_{k-1},A_{k}^{}\right)+N\left(0,\Sigma_{k}^{INS}\right)$$

(36) $${\left\|e_{k}^{INS}\right\|}_{\Sigma_{k}^{INS}}^{2}={\left\|x_{k}-h^{INS}\left(x_{k-1},A_{k}^{}\right)\right\|}_{\Sigma_{k}^{INS}}^{2}$$

where $h^{INS}\left(\cdot \right)$ denotes the measurement function, $A_{k}^{}$ denotes the IMU measurements, and $\Sigma_{k}^{INS}$ denotes the covariance matrix. 

### 3.2. GNSS Factor

Similarly, the cost function for GNSS measurement is written as

(37) $${\left\|e_{k}^{GNSS}\right\|}_{\Sigma_{k}^{GNNS}}^{2}={\left\|z_{k}^{GNSS}-h^{GNSS}\left(x_{k}\right)\right\|}_{\Sigma_{k}^{GNSS}}^{2}$$

where $z_{k}^{GNSS}$ denotes the GNSS measurements, $h^{GNSS}\left(\cdot \right)$ denotes the measurement function, and $\Sigma_{k}^{GNSS}$ denotes the covariance matrix of the measurement noise. 

### 3.3. NHC Factor

As the measurement model listed in Equations (9)–(11), the cost function of the NHC measurement is written as

(38) $${\left\|e_{k}^{NHC}\right\|}_{\Sigma_{k}^{NHC}}^{2}={\left\|z_{k}^{NHC}-h^{NHC}\left(x_{k}\right)\right\|}_{\Sigma_{k}^{NHC}}^{2}$$

where $z_{k}^{NHC}$ denotes the NHC measurements, $h^{NHC}\left(\cdot \right)$ denotes the measurement function, and $\Sigma_{k}^{NHC}$ denotes the covariance matrix of the measurement noise. 

### 3.4. Odometer Factor

The cost function of the odometer measurement is written as

(39) $${\left\|e_{k}^{Odo}\right\|}_{\Sigma_{k}^{NHC}}^{2}={\left\|z_{k}^{Odo}-h^{Odo}\left(x_{k}\right)\right\|}_{\Sigma_{k}^{Odo}}^{2}$$

where $z_{k}^{Odo}$ denotes the odometer measurements, $h^{Odo}\left(\cdot \right)$ denotes the measurement function, and $\Sigma_{k}^{Odo}$ denotes the covariance matrix of the measurement noise. 

### 3.5. FGO

Optimal estimates of the states are obtained through minimizing the cost functions, which is formulated as

(40) $$x^{*}=\arg\min\Sigma_{k}\left({\left\|e_{k}^{INS}\right\|}_{\Sigma_{k}^{INS}}^{2}+{\left\|e_{k}^{GNSS}\right\|}_{\Sigma_{k}^{GNSS}}^{2}\right)$$

Under GNSS-denied conditions, the optimal states in the FGO-IMU/NHC/Odometer are solved through the formulations listed as

(41) $$x^{*}=\arg\min\Sigma_{k}\left({\left\|e_{k}^{NHC}\right\|}_{\Sigma_{k}^{NHC}}^{2}+{\left\|e_{k}^{Odo}\right\|}_{\Sigma_{k}^{NHC}}^{2}+{\left\|e_{k}^{INS}\right\|}_{\Sigma_{k}^{INS}}^{2}\right)$$

## 4. Experiments and Results

To assess the performance of the proposed method, we carried out two different experiments. Raw GNSS, IMU, and odometer measurements were collected through our data-collecting platform installed in a ground vehicle. Our previous paper revealed the mode details of the MEMS IMU and data collecting [37], Table 1 lists the specifications of the employed IMU in this experiment. Here, we first assess the FGO-based GNSS/INS performance for ground vehicle position applications; then, FGO-INS/NHC/Odometer integration is evaluated with the collected raw data. We utilize a postprocessing GNSS/INS reference system POS320 as the trajectory reference for the experiments to calculate the position errors.

### 4.1. FGO-GNSS/INS Performance Assessment

**Figure 3:**
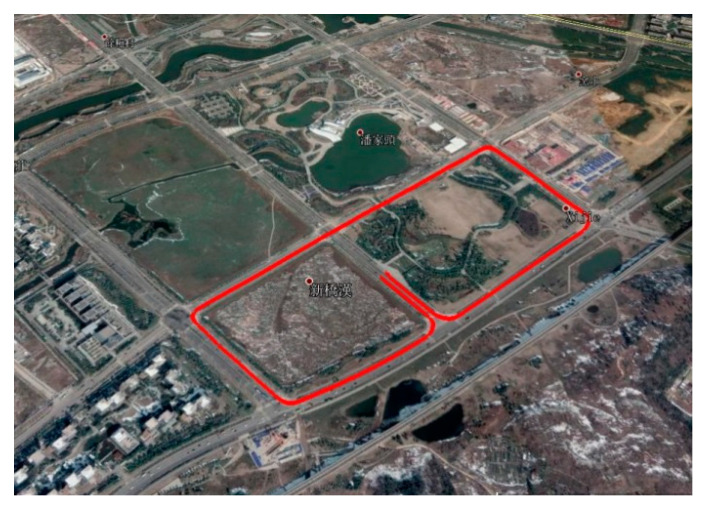
The trajectory of the experiment.

**Figure 4:**
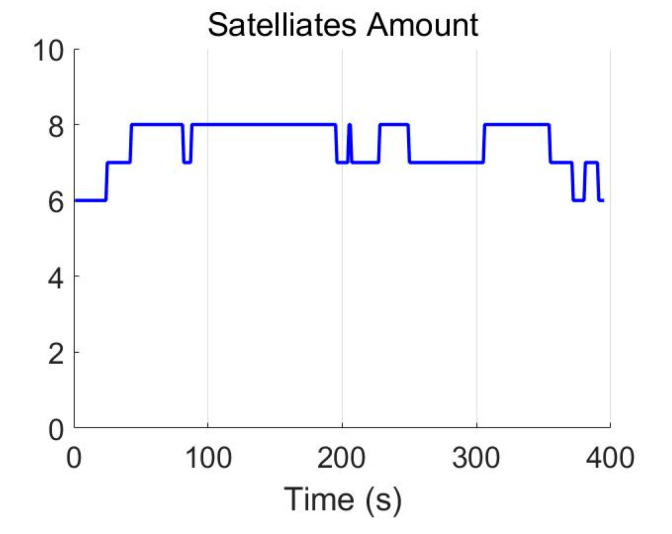
Satellite amount.

**Figure 5:**
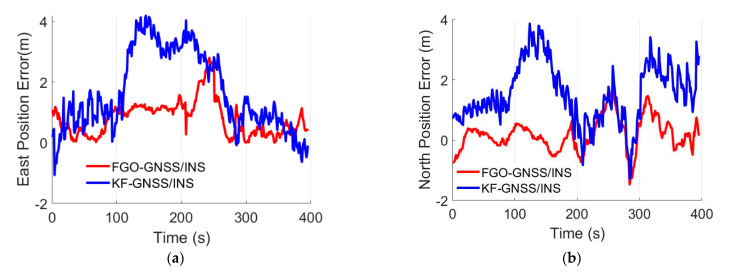

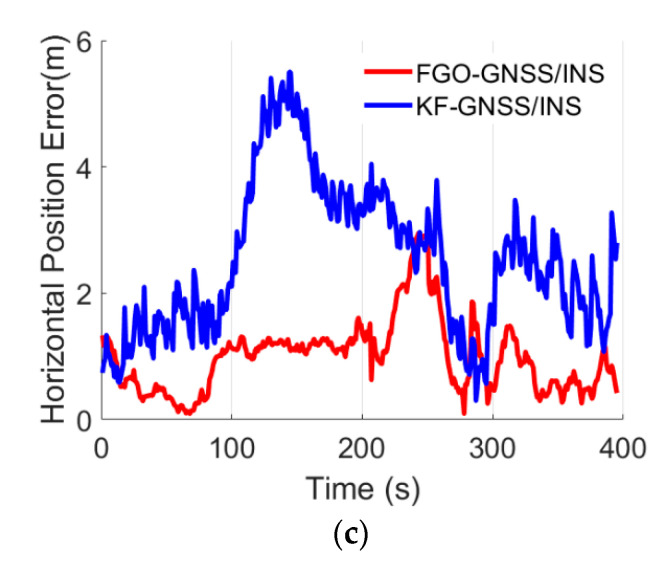
Position errors comparisons. (**a**) East-direction position error. (**b**) North-direction position error. (**c**) Horizontal position errors.

We carried out the field test near the Nanjing University of Science and Technology. Figure 3 presents the trajectory plotted in the Google Map. Figure 4 shows the available satellites for the experiment. It can be seen that the amount of the in-view satellites is satisfactory. Position errors from the GNSS/INS integration methods KF-GNSS/INS and FGO-GNSS/INS are presented in Figure 5 together for comparison. Specifically, Figure 5a shows the east-direction position errors comparison, Figure 5b illustrates the north-direction position errors comparison, and Figure 5c presents the horizontal position errors comparison. Blue curves represent the position errors of the KF-GNSS/INS method, and the red curves represent the position errors of the FGO-GNSS/INS method. It can be observed that the FGO-GNSS/INS obtains superior performance compared with KF-GNSS/INS through the position curves. 

**Figure 6:**
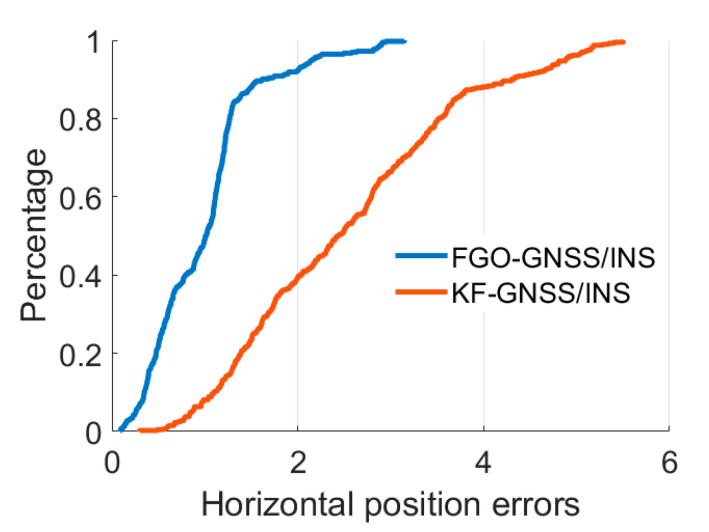
Cumulative distribution function (CDF) for the horizontal position errors.

Furthermore, the quantitative analysis results of the absolute values of the position errors are listed in Table 2. In terms of the mean values of the absolute position errors, the FGO-GNSS/INS obtains a 52.6%, 46.8%, and 60.7% decrease for east, north, and horizontal position errors compared with that of the KF-GNSS/INS. Regarding the rooted mean square (RMS) position errors, the FGO-GNSS/INS also obtains a 53.6%, 49.5%, and 51.3% decrease. Additionally, the maximum values of the east, north, and horizontal position errors from the FGO-GNSS/INS decreased by 33.1%, 36.2%, and 42.7% compared with that from the KF-GNSS/INS. Furthermore, Figure 6 shows the distribution of the horizontal position errors, and it also supports that the FGO-GNSS/INS has superior performance.

**Figure 7:**
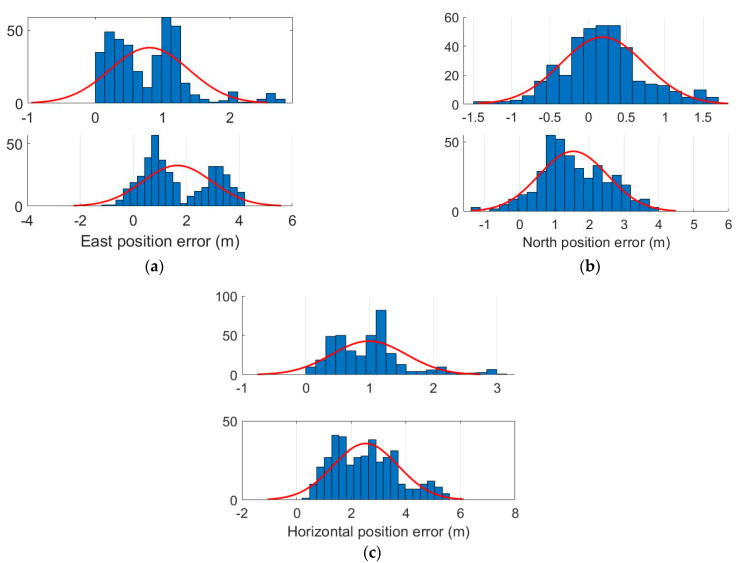
Position errors distribution. (**a**) East-position errors distribution. (**b**) North-position errors distribution. (**c**) Horizontal-position errors distribution.

Here, we assumed the position errors are subject to Gaussian distribution, and we analyzed the position errors distribution. Figure 7 presents the distributions of these position errors. In this experiment, the GNSS receiver was developed by us with DSP + FPGA and C++ programming language. It seems that the position errors are not extremely subject to Gaussian distribution. In Figure 7, the upper part of each figure shows the FGO-GNSS position errors distribution, and the bottom part represents the KF-GNSS/INS position errors distribution. It can be observed that the mean values of the FGO-GNSS/INS are decreased, and the corresponding position errors distribution range is reduced.

### 4.2. FGO-INS/NHC/Odometer under GNSS-Denied Environments

**Figure 8:**
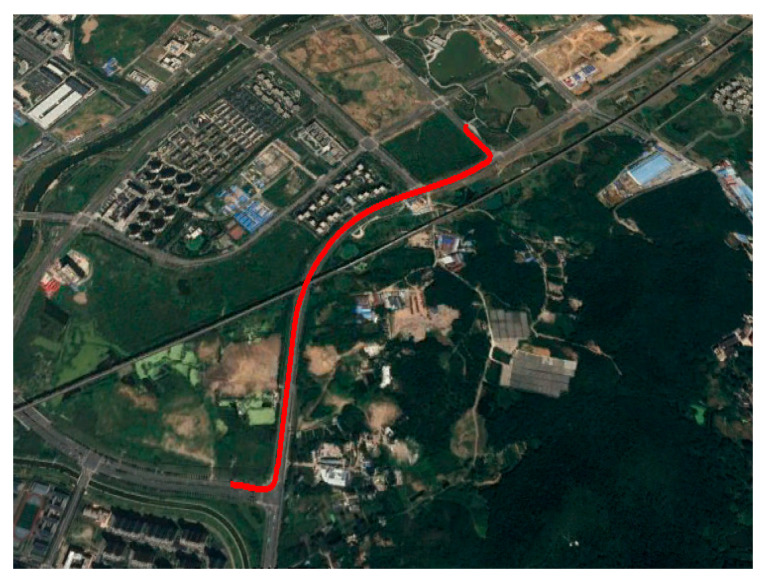
Field test trajectory.

**Figure 9:**
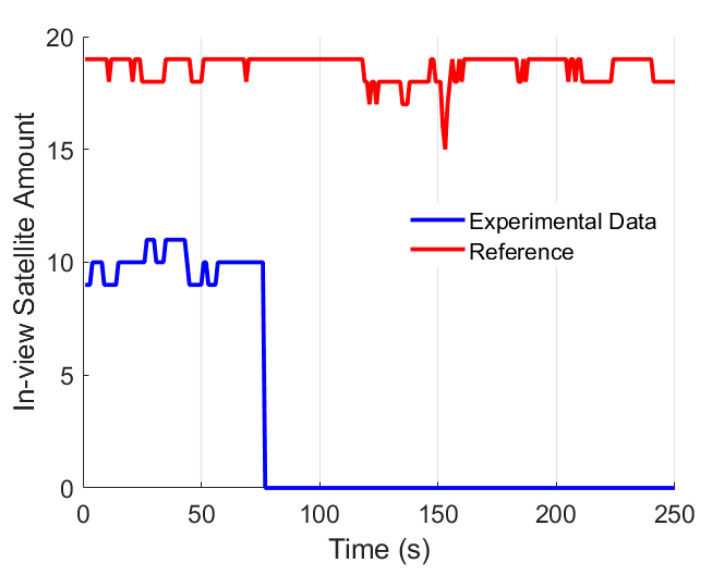
Satellite’s amount [37].

We collected the raw measurements using the exact same ground vehicle in the second field test. The testing trajectory is presented in Figure 8, and the in-view satellite’s amount during the testing is shown in Figure 9. In Figure 9, the red line represents the in-view satellite’s amount of the reference, and the blue line represents the in-view satellite’s amount of our system. We simulated the GNSS-denied environment by removing the antenna from the GNSS receiver. Here, at approximately 75 s, we remove the GNSS antenna, and the available satellite’s amount immediately decreases to zero. Here the dataset is the same as our previously published paper [37]. 

**Figure 10:**
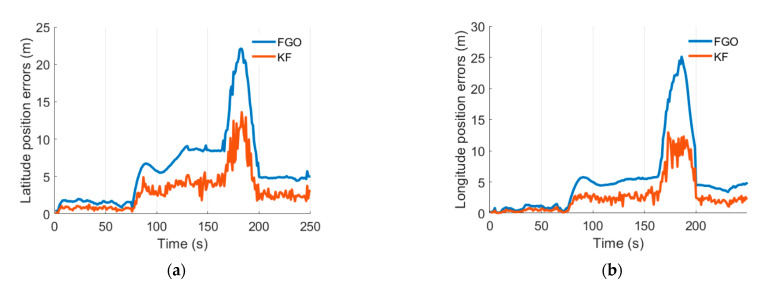
Position errors. (**a**) Latitude errors. (**b**) Longitude errors.

**Figure 11:**
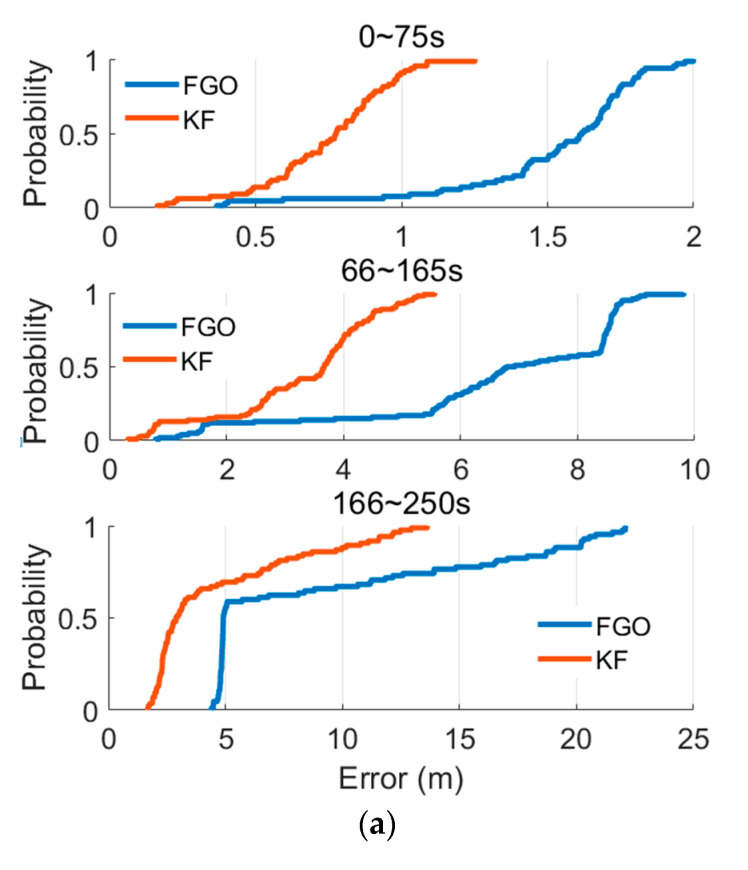

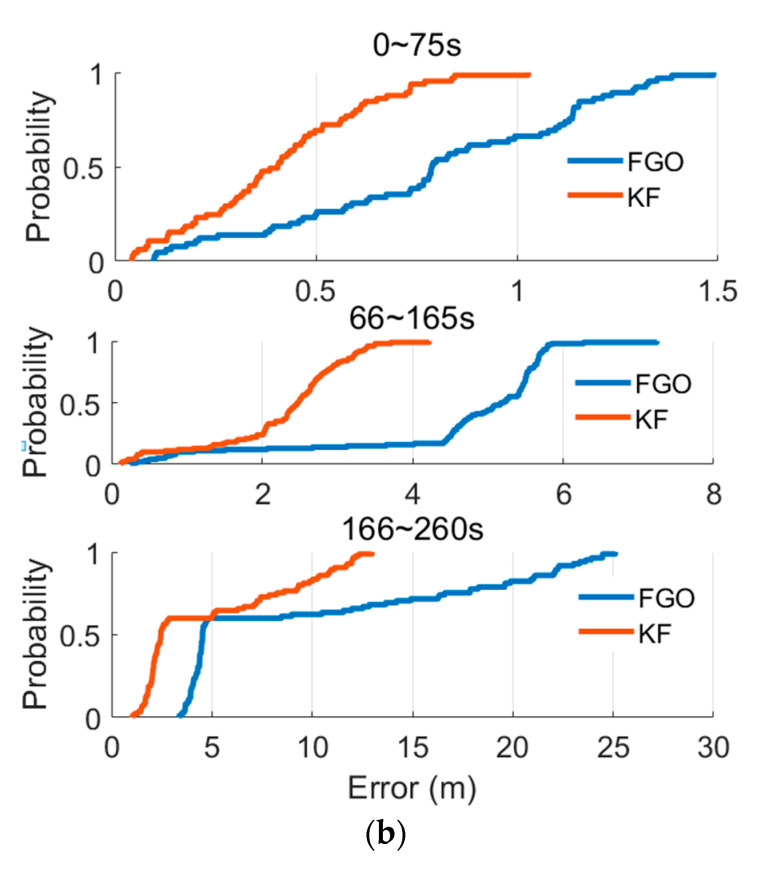
Position errors distribution. (**a**) Latitude errors distribution. (**b**) Longitude-position errors distribution.

Latitude and longitude position errors are presented in Figure 10. The red lines represent the position errors from the KF method, and the blue lines represent the position errors from the FGO method. During 0~75 s, the available satellites are regular, the system works under GNSS/INS integration system, and the latitude and longitude errors are smaller than five meters. Under this model, the FGO method slightly decreases the positioning errors. Table 3 and Table 4 illustrate the latitude and longitude position errors during different periods. Specifically, the mean values of the latitude errors decreased by 50.7%, and the mean values of the longitude errors decreased by 49.4%; also, the maximum values of the position errors decreased by 37.2% and 30.9% for latitude and longitude position errors. Then, after 75 s, the GNSS antenna is removed from the system to simulate the GNSS-denied environment. The latitude and longitude position errors from the KF and FGO methods increase. Moreover, the FGO method brings a noticeable decrease in positioning errors. Specifically, the mean values of the latitude errors decreased by 50.1%, and the mean values of the longitude errors decreased by 50.4%; the maximum values of the latitude and longitude position errors decreased by 43.3% and 41.8%. The system works under IMU/odometer/NHC integration mode. After the 165th second, the odometer was excluded from the system, and the system operated under IMU/NHC mode, so positioning errors increased further. The latitude and longitude errors from the KF-IMU/NHC integration are more significant than 20 m. FGO-IMU/NHC integration decreases compared with the KF- IMU/NHC. Specifically, compared with the KF method, the mean values of the latitude errors from the FGO method decreased by 49.5%, and the mean values of the longitude errors from the FGO method decreased by 50.2%; the maximum values of the latitude and longitude position errors from the FGO method decreased by 38.3% and 48.3%. In addition, the cumulative distribution function (CDF) results of these position errors are presented in Figure 11; it is evident that the FGO method contributes to a better distribution of the position errors, including more minor mean and maximum position errors.

## 5. Limitations and Discussion

The experimental results reveal that the FGO-INS/NHC/Odometer integration can contribute to better outcomes for the KF-INS/NHC/Odometer during signal outage. However, we think there are still the following limitations:

(1) in the paper, the optimization is conducted using all the past information, and the computation load increases exponentially. Now, it is still hard to implement the FGO in a real-time manner; it is helpful to reduce the computation load with a fixed smoothing window.
(2) the measurement noises are subject to Gaussian distribution, as the position errors’ distribution plotted in Figure 7 is not strictly subject to standard Gaussian distribution. In addition, due to the environmental influence, the GNSS or INS measurement errors covariance matrix might be changed; it was more feasible for adaptively tuning the errors matrix in the FGO method; in fact, some adaptive KFs have been proposed and demonstrated in dealing with this problem, and some strategies could be adopted and utilized in FGO.
(3) in urban areas, GNSS measurements might face abnormal measures induced by the multipath or non-line-of-sight (NLOS) signals; it is adequate to add some kernel functions to the FGO to mitigate the adverse effects and improve the robustness of the navigation solutions.

## 6. Conclusions

Improving the position accuracy for ground vehicles under GNSS-signal-challenging environments is a hot research topic in the community, this paper investigated FGO-GNSS/INS and FGO-INS/NHC/Odometer integration, especially for GNSS signal outages. Two different field experiments were carried out. With analyzing the experimental results, the following findings are concluded: (1) the FGO method is effective for improving the accuracy of the INS navigation solutions compared with that from KF; (2) the FGO method can improve the position accuracy without GNSS compared with KF. This work preliminarily demonstrates that FGO is a better senor fusion method for GNSS/INS integration applications compared with KF. FGO is a feasible method to improve position accuracy under GNSS signal challenging environments. Actually, with the FGO framework, more sensors can be added to the fusion framework to obtain a better position result. For instance, a LiDAR or visual camera can be easily integrated with GNSS and INS, which could adaptively select proper measurements from sensors to compose a feasible integration system and obtain the most optimal navigation solutions. 

## Figures and Tables

**Table 1:** Inertial measurement unit (IMU) specifications [37].

**Gyroscope**	**Bias stability (degree/h)**	≤3 degree/h
	**Scale factor nonlinearity (ppm)**	≤200 ppm
	**White noise (degree/h)**	0.1 degree/h
**Accelerometer**	**Bias stability (mg)**	0.1 mg
	**Scale factor nonlinearity (ppm)**	≤150 ppm
	**White noise (mg)**	0.05 mg

**Table 2:** Latitude position errors analysis and companions.

	KF			GO		
	East	North	Horizontal	East	North	Horizontal
Mean (m)	1.71	1.59	2.53	0.81	0.86	0.99
RMS (m)	1.25	0.91	1.19	0.58	0.46	0.58
Maximum (m)	4.20	3.87	5.50	2.81	2.47	3.15

**Table 3:** Latitude-position errors analysis and companions.

	KF		FGO	
Time (s)	Mean (m)	Maximum (m)	Mean (m)	Maximum (m)
**0~75 s**	1.50	1.99	0.74	1.25
**76~165 s**	7.16	9.81	3.57	5.56
**166~250 s**	9.14	22.09	4.62	13.62

**Table 4:** Longitude-position errors analysis and companions.

	KF		FGO	
Time (s)	Mean (m)	Maximum (m)	Mean (m)	Maximum (m)
**0~75 s**	0.77	1.49	0.39	1.03
**76~165 s**	5.04	7.25	2.50	4.22
**166~250 s**	9.87	25.10	4.92	12.98

## Data Availability

Not applicable.
